# Adaptive token division-based transformer for visual object tracking

**DOI:** 10.1371/journal.pone.0351112

**Published:** 2026-08-07

**Authors:** Dan Tian, Dongxin Liu, Xiao Wang

**Affiliations:** School of Intelligent Science and Information Engineering, Shenyang University, Shenyang, Liaoning, China; International Institute of Information Technology Bangalore, INDIA

## Abstract

In visual object tracking, one-stream trackers typically use all search tokens to interact with templates across all encoder layers. However, the search area usually contains a lot of interference information, such as distractors with similar appearance to the tracking object, which will cause the distractors in the search area to be misjudged as interactive objects, establish wrong cross-relation modeling, and reduce the accuracy of tracking. To alleviate this issue, this paper proposes a transformer-based visual object tracking framework with adaptive token division. First, our tracking framework is a simple encoder-decoder structure without any post-processing. Second, we propose an adaptive token division module, which enables search tokens and template tokens to perform the most appropriate cross-relationship modeling, and improves the model ‘s ability to distinguish between object and background. At the same time, we introduce an attention masking strategy and Gumbel-Softmax technique. The strategy enables efficient and parallel attention calculation between different categories of tokens, and the technique facilitates the end-to-end optimization of the division module. Finally, we conduct tests on six tracking benchmarks, and the experimental results prove the effectiveness of our method.

## 1. Introduction

Visual object tracking [1] is an important research content in the field of computer vision, which aims to locate a specific object and track its motion trajectory in real time within consecutive video sequences. With the rapid development of artificial intelligence technology, visual object tracking has shown a wide range of application prospects in many fields such as autonomous driving, intelligent monitoring, and human-computer interaction [2].

At present, the mainstream visual object tracking methods are mainly divided into two-stream [3–5] and one-stream [6–8]. These methods typically treat the tracking problem as a per-frame template matching task, generally divided into three stages: (i) feature extraction from both search and template images; (ii) feature matching and fusion using convolutional or attention mechanisms; (iii) use customized head networks to perform operations such as center point localization and scale estimation. Compared with the two-stream methods, the one-stream methods have a simpler network structure and higher computational efficiency. The one-stream methods leverage attention mechanisms to jointly extract visual features and model relationships between template regions and search regions. However, the use of a customized head network increases the complexity of the network framework and may require additional training. SeqTrack [9] and ARTrack [10] modeled tracking as a sequence generation problem. They use the encoder-decoder transformer structure, which does not need to design complex head networks and simplifies the network structure.

References [7,8,11] indicate that early interaction between the template and the search region facilitates the generation of features with greater object discrimination. However, not all parts within the search region are suitable for interaction with the template. In fact, the search region usually contains a lot of interference information, such as interferences similar to the appearance of the tracking object, which will lead to the interference in the search area being misjudged as interactive objects, establish wrong cross-relationship modeling, and reduce the accuracy of tracking.

Although attention mechanisms can suppress inappropriate feature interactions to some extent, the application of global interaction modeling to all encoder layers inevitably introduces some interference. Specifically, this interference is mainly reflected in two aspects: First, when the distractor in the search region interacts incorrectly with the template, the distractor may aggregate the object features, which will make it difficult to locate the actual object in the search region accurately. Second, in the process of feature updating, improper interaction between the template tokens and distractors in the search region will reduce the quality of the template feature. The above situation will weaken the ability of the one-stream tracking methods to distinguish between the object and the background, which reduces the accuracy of the algorithm.

To address the above issues, we propose a transformer-based visual tracking framework with adaptive token division, which only uses the encoder-decoder transformer architecture. By introducing the adaptive token division module into the transformer encoder, the search tokens and the template tokens can be used to model the most suitable cross-relationship, suppress the influence of background information or similar interferences, and improve the model ‘s ability to distinguish between object and background. Specifically, we divide all tokens into three categories: one is template tokens, and the other two are different types of search tokens. Among them, only the search tokens judged as the tracking object can interact with the template tokens, and the search tokens judged as the background cannot interact with the template tokens.

The categorization of search tokens relies on the adaptive token division module, but two problems need to be solved in the actual design: one is how to realize the interaction between different token categories efficiently and parallelly, and the other is how to ensure the end-to-end optimization of discrete token classification. For the first issue, we use the attention masking mechanism to integrate the original multiple attention operations into a unified attention operation. For the second issue, we introduce the Gumbel-softmax technique [12] to make the discrete classification process differentiable. In addition, compared with [9] and [10], our model adopts a similar transformer structure, only adds an adaptive token division module to the encoder. This paper makes three main contributions:

(1) We design an adaptive token division module and introduce it into the transformer encoder, so that the search tokens and the template tokens can be used for the most appropriate cross-relationship modeling. This can improve the model ‘s ability to distinguish between object and background. Meanwhile, we use a simple encoder-decoder structure without designing complex head networks for post-processing.
(2) We introduce the attention masking mechanism and Gumbel-softmax technique to enable efficient and parallel interaction between different categories of tokens, and facilitate end-to-end optimization of the adaptive token division module.
(3) Compared to other state-of-the-art trackers, our method achieves more advanced performance on datasets such as Lasot, GOT-10k, and TrackingNet.

## 2. Related works

### 2.1. Visual tracking framework

In the past, object tracking methods based on siamese network architecture [13–16] have made some progress in the field of visual tracking. Such methods usually use a two-branch structure to extract features from the template and the search region respectively, and then perform feature fusion through specific modules. With the rapid development of transformer in the field of computer vision, it has shown excellent performance in object tracking tasks. Consequently, numerous state-of-the-art trackers [2–6,17,18] now adopt transformers as their core architecture.

In recent years, one-stream tracking frameworks [6–8] have used self-attention mechanisms for joint feature extraction and relationship modeling, which has effectively enhanced their feature interaction capability. Although these one-stream methods are different from the previous two-stream methods in structure, they all treat the search area as a whole, that is, the template features interact with the entire search area. However, not all parts of the search area are suitable for interacting with the template. When the feature expression ability is insufficient, it is easy to cause confusion between the object and the background information. To solve this problem, we propose an adaptive token division module. This model enables search tokens and template tokens to establish the most appropriate cross-relationship modeling in each encoder layer, which helps suppress background interference.

In addition, these methods usually need to design a specific head network for post-processing operations such as object location and scale estimation, which increases the complexity of the model. The design of the head network also needs to introduce multiple loss functions, and more hyperparameters need to be adjusted, which increases the difficulty and uncertainty of model tuning. To this end, SeqTrack [9] and ARTrack [10] model tracking as a sequence generation task. They adopt the encoder-decoder transformer structure, and do not need to design a specific head network and multiple loss functions. Our model uses a similar transformer structure, only adding an adaptive token division module to the encoder.

### 2.2. Tracking based on dynamic design

Some tracking algorithms achieve diversity objectives by introducing dynamic design at different stages of the model. LightTrack [19] and AutoMatch [20] leverage neural architecture search techniques to dynamically optimize network structures during training. LightTrack focuses on searching lightweight backbone network and prediction head architecture. This goal is to achieve a balance between computational efficiency and tracking accuracy. AutoMatch focuses on automatically designing diverse matching operators to enhance the robustness of related modules. However, the network structures of these methods are fixed once training is completed. Consequently, they lack the ability to dynamically adjust based on input information during inference. In contrast, our method supports dynamic adjustments during inference. Through the adaptive token division module, it dynamically selects the most suitable search tokens for interaction with the template in each encoder layer. As a result, our approach offers significantly greater flexibility.

To optimize the performance of visual transformers, researchers [21–23] have proposed various token organization strategies. These approaches fully leverage the high flexibility of attention mechanisms. SparseTT [24] employs a sparse attention mechanism. Through this method, the model can selectively focus on the most similar token features, which enhances foreground-background discrimination. OSTrack [8] designs an early candidate elimination module. With this module, the model can remove irrelevant tokens in the search region. This significantly improves computational efficiency. GRM [25] uses a lightweight prediction module to classify input tokens, which enables flexible relationship modeling. Although these methods have achieved excellent performance, their structures are complex. They need to design specialized head networks for subsequent object localization.

To address the above problems, this paper proposes a transformer-based visual tracking framework with adaptive token division, which only uses encoder and decoder without any post-processing. We integrate an adaptive token division module into the encoder. This allows flexible interaction between search and template tokens, suppresses interference from irrelevant information in the search region, and achieves more accurate tracking.

## 3. Method

In this section, we present a detailed description of the proposed method. First, we briefly outline the algorithmic framework. Then, we describe the model architecture in detail. Finally, we introduce the training and inference strategies.

### 3.1. Overview

**Fig 1:**
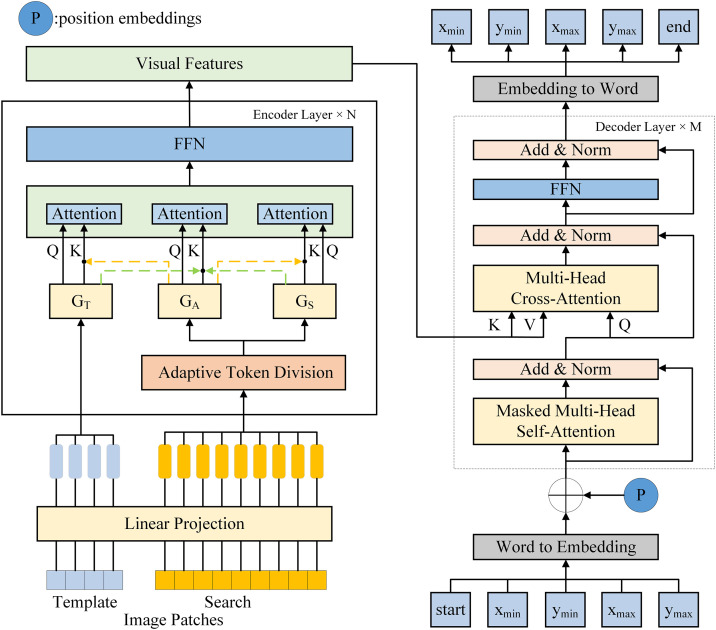
Overall architecture of our method. The encoder is responsible for extracting visual features, while the decoder uses the extracted visual features to generate the final prediction sequence. Through the proposed attention masking strategy, three parallel attention calculations are performed. For simplicity, we only mark the queries and keys in parallel attention computing. The values are identical to the keys and are omitted here.

Our tracker framework, as shown in Fig 1, adopts an encoder-decoder transformer architecture. First, both the search image and template images are divided into image patches. These patches are then transformed into visual embeddings via linear projection and fed into the encoder. The input image is divided into three categories by the adaptive token division module, where G_A_ represents the object tokens in the search region, G_S_ represents the remaining tokens in the search region, and G_T_ is all template tokens. The visual features are output through three parallel attention calculations. During training, the decoder takes [start, x_min_, y_min_, x_max_, y_max_] as the input sequence and [x_min_, y_min_, x_max_, y_max_, end] as the output sequence. Here, [x_min_, y_min_, x_max_, y_max_] is converted from the object bounding box, and the start and end tokens control the start and end of the sequence generation task, respectively. During inference, the input sequence begins with [start], and newly generated bounding box tokens are appended sequentially at each step. The inference task concludes once all four bounding box tokens are generated. To ensure that each token can only focus on the previous information during its generation, a causal attention mask is applied to the self-attention module of the decoder.

### 3.2. Image and sequence representation

The input of the encoder includes a search image $x\in R^{3\times H_{x}\times W_{x}}$ and a template image $z\in R^{3\times H_{z}\times W_{z}}$. Before inputting the image into the encoder, the search image and the template image are first divided into image patches $x_{p}\in R^{N_{x}\times \left(3\times P^{\text{2}}\right)}$ and $z_{p}\in R^{N_{z}\times \left(3\times P^{\text{2}}\right)}$, where $N_{x}=H_{x}W_{x}/P^{\text{2}}$ and $N_{\text{z}}=H_{\text{z}}W_{\text{z}}/P^{\text{2}}$ represents the number of image patches, and P×P is the size of the image patch. Then, the image patch is converted into visual embeddings $G_{x}\in R^{N_{x}\times C}$ and $G_{z}\in R^{N_{z}\times C}$ by linear projection, where C is the embedding dimension. Finally, the visual embeddings are input to the encoder for subsequent processing.

The input sequence of the decoder is [start, x_min_, y_min_, x_max_, y_max_], which is converted from the object bounding box. We discretize all continuous coordinate values and map them into a unified vocabulary V. Each coordinate value is converted into integers between [1, n], which are considered as words in vocabulary V. Specifically, each value in the input sequence and the object sequence (i.e., the coordinate value of the bounding box) corresponds to a word in the vocabulary. During training, the model learns how to generate the object sequence from the input sequence. During inference, the model maps the generated integers to coordinate values from its vocabulary, and finally outputs the prediction of the bounding box.

### 3.3. Model architecture

#### 3.3.1. Encoder.

The encoder’s structure is the same as the vision transformer [26], except for one modification: an adaptive token division module is added to encoder layers. First, both the search and template images are divided into image patches. These image patches are converted into visual embeddings through linear projection, and then fed into the encoder. Second, the input tokens are divided into three categories: G_T_, G_A_ and G_S_ by using the adaptive token division module. G_T_ can aggregate the tokens information in G_T_ and G_A_, G_S_ can aggregate the tokens information in G_S_ and G_A_, and G_A_ can aggregate all the tokens information. Finally, the visual features are output through three parallel attention calculations.

#### 3.3.2. Decoder.

Each decoder layer consists of masked multi-head self-attention, multi-head cross-attention, and a feedforward network. The word-to-embedding layer converts the input sequence into word embeddings, adds positional information, and feeds it into the decoder’s masked multi-head self-attention layer. Due to the masking effect, the generation of each sequence element depends solely on its preceding elements. Subsequently, multi-head cross-attention fuses the visual features extracted by the encoder with the word embeddings. Finally, the feedforward network layer feeds the output of the multi-head cross-attention into the next decoder block.

#### 3.3.3. Adaptive token division module.

In order to adaptively divide the search tokens into G_S_ and G_A_, we propose an adaptive token division module and add it to encoder layers. Specifically, to provide object-related context information to search tokens, we first aggregate all template tokens through a global maximum pooling. This operation will generate an object-aware global representation. Subsequently, the object-aware representation is concatenated with the features of each search token. Finally, the concatenated features are processed by a lightweight multi-layer perceptron (MLP). This MLP predicts classification probabilities for each search token. The outputs represent the likelihood of belonging to G_S_ and G_A_:


$$\begin{array}{c} \pi_{i}=Softmax\left(MLP\left(\left[MaxPool\left(G_{z}\right);G_{x}^{i}\right]\right)\right)=\left[\pi_{i,0},\pi_{i,1}\right]\in R^{N\times 2} \end{array}$$ (1)

Where $G_{z}$ and $G_{x}^{i}$ denote the template tokens embedding and the i-th search token embedding respectively, $\pi_{i,0}$ and $\pi_{i,1}$ denote the probability that the i-th search token belongs to G_S_ and G_A,_ respectively. Based on the predicted values $\pi_{i,0}$ and $\pi_{i,1}$, the model selects the larger probability to determine the search token’s category. Although it seems easy to apply this module for search token division, two obstacles need to be solved. That is how to achieve efficient parallel attention computing, and how to achieve end-to-end optimization learning of this module.

The first issue stems from how to model the cross-relations of the three token categories. The number of queries and keys in these three categories is different, which makes it difficult to perform three attention calculations in parallel. This will reduce the tracking speed. To address this, we design an attention masking strategy. Specifically, we first convert the probability π into one-hot encoding $Q\in {\left\{0,\;1\right\}}^{N_{x}\times 2}$. Then we define two one-hot tensors: $Q_{z}\in {\left\{0,\;1\right\}}^{N_{z}\times 3}$ denotes the category assignment of template tokens, and $Q_{x}\in {\left\{0,\;1\right\}}^{N_{x}\times 3}$ denotes the category assignment of search tokens. In $Q_{z}$, the first column is all set to 1, and the other two columns are set to 0, indicating that all template tokens are divided into G_T_. $Q_{x}$ is obtained by adding a column of zeros before Q, which ensures that the search tokens are not categorized into G_T_. Finally, the merged $\mathrm{\backslash stackrel}\wedge Q=\left[Q_{z};Q_{x}\right]\in {\left\{0,\;1\right\}}^{\left(N_{z}+N_{x}\right)\times 3}$ is used to construct the attention mask $M\in {\left\{0,\;1\right\}}^{\left(N_{z}+N_{x}\right)\times \left(N_{z}+N_{x}\right)}$, where the value $M_{i\text{,}\;j}$ of each element is calculated as follows:


$$\begin{array}{c} M_{i\text{,}\;j}={\mathrm{\backslash stackrel}\wedge Q}_{i,0}({\mathrm{\backslash stackrel}\wedge Q}_{j,0}+{\mathrm{\backslash stackrel}\wedge Q}_{j,2})+{\mathrm{\backslash stackrel}\wedge Q}_{i,1}({\mathrm{\backslash stackrel}\wedge Q}_{j,1}+{\mathrm{\backslash stackrel}\wedge Q}_{j,2})+{\mathrm{\backslash stackrel}\wedge Q}_{i,2}({\mathrm{\backslash stackrel}\wedge Q}_{j,0}+{\mathrm{\backslash stackrel}\wedge Q}_{j,1}+{\mathrm{\backslash stackrel}\wedge Q}_{j,2}) \end{array}$$ (2)

The value of $M_{i\text{,}\;j}$ determines whether token i can aggregate information from token j. By element-wise multiplying this attention mask matrix M with the original complete attention weight matrix, the attention weight of the interactive position prohibited will be set to zero. We can effectively merge the original three independent attention calculations into a single calculation, and then achieve parallel attention calculation.

The second issue is that the classification of search tokens is essentially a discrete decision-making process. This discrete operation is non-differentiable, which prevents gradient calculation during backpropagation. As a result, the parameters of the division module cannot be directly optimized through gradient descent. To address this, we introduce the Gumbel-Softmax technique, which adds Gumbel distribution noise to the classification probability π, so that the sampling process becomes differentiable:


$$\begin{array}{c} Q=One\text{-}hot\left(\underset{i}{argmax}\left[g_{i}+log\pi_{i}\right]\right)\in {\left\{0,\;1\right\}}^{N_{x}\times 2} \end{array}$$ (3)

Where g~Gumbel(0,1). The Q is used for the classification of search tokens in forward propagation. In the back propagation, the softmax operation with temperature parameters is used to approximate the argmax operation:


$$\begin{array}{c} y_{i}=\frac{exp\left(\left(log\left(\pi_{i}\right)+g_{i}\right)/\tau \right)}{\sum_{j}^{N_{x}}exp\left(\left(log\left(\pi_{j}\right)+g_{j}\right)/\tau \right)} \end{array}$$ (4)

where τ represents the temperature parameter and $y_{i}$ represents the gradient. The gradient is calculated by the continuous output of softmax, so that the adaptive division module can achieve end-to-end optimization. For a complete derivation of this technique, please refer to reference [12].

### 3.4. Training and inference

#### 3.4.1. Training.

During training, the adaptive token division module calculates the category probability π for each search token, then uses Formula (3)to generate one-hot encoding Q, so as to construct the attention mask matrix M and realize parallel attention calculation. In the back propagation, the model uses the softmax approximation in Formula (4)to calculate the gradient, and combines the loss function to realize the end-to-end optimization of the division module.

We use the cross-entropy loss function for training, which aims to maximize the log-likelihood between the generated object sequence and the actual object sequence. The loss function for the training process is as follows:


$$\begin{array}{c} maximize\sum_{j=1}^{L}logP\left(s_{j}|x,z,s_{<j}\right) \end{array}$$ (5)

Where P(·) represents the probability of softmax, x and z represent the search image and the template image respectively, $s_{j}$ represents the currently predicted object sequence, j represents the position of the currently generated token in the sequence, $s_{<j}$ represents the object sequence before j, and L represents the length of the object sequence.

#### 3.4.2. Inference.

During inference, the adaptive token division module performs a binary classification of each search token, and directly takes argmax to generate one-hot encoding Q without Gumbel noise. The input sequence of the decoder is [start], which marks the beginning of the task. Then, the model generates the object bounding box sequence [x_min_, y_min_, x_max_, y_max_, end] in an autoregressive manner. In each generation step, the model is sampled from the vocabulary V based on the maximum likelihood, that is, $s_{j}={argmax}_{v_{j}}P\left(v_{j}|x,z,s_{<j}\right)$, where $v_{j}$ is the word in the vocabulary V.

## 4. Experiments

### 4.1. Implementation details

The encoder of our model adopts ViT-B [26] architecture, which is pre-trained using the MAE [27] model. The resolution of the search image and the template image is set to 256 × 256 and 128 × 128, respectively, and the size of the image patch is 16 × 16. The decoder layer is set to 2, the attention head is set to 8, the dropout rate is 0.1, and the FFN hidden size is 1024. The vocabulary size is set to 4000, and both the word embedding dimension and the decoder’s hidden dimension are set to 256. All adaptive token division modules adopt the same structure, that is, a lightweight MLP, which consists of two hidden layers that use the GELU activation function [28] and a final output layer. Their channel sizes are 384, 192, and 2, respectively.

The training datasets include COCO [29], LaSOT [30], GOT-10k [31], and TrackingNet [32]. According to the official requirements, we remove the 1k video sequences prohibited in the GOT-10k dataset during training. We use brightness jitter and horizontal flip to achieve data enhancement. The entire model is optimized for 300 epochs using the AdamW optimizer [33]. The learning rate for the backbone network is set to 4 × 10^−5^, while for other parameters it is set to 4 × 10^−4^. After 240 epochs, the learning rate is reduced by a factor of 10. For the GOT-10k dataset, we train for 150 epochs, with the learning rate decreased by a factor of 10 after 120 epochs. Our experiments are performed on Intel Core i9-13900KF CPU @ 3.00GHz, 32GB RAM and a single GeForce RTX4090 GPU. The running speed of our tracker is about 65 fps, which is an average value obtained by directly performing continuous inference on a 1000-frame long video using a single GeForce RTX4090 GPU.

### 4.2. Main results and comparisons

We compare our method with some state-of-the-art trackers on six tracking benchmarks: LaSOT [30], GOT-10k [31], TrackingNet [32], UAV123 [34], NFS [35], and TNL2K [36].

LaSOT is a large-scale benchmark dataset designed for single object tracking tasks. It contains a total of 1,400 video sequences. The average length of each sequence is about 2,500 frames, and the total number of frames exceeds 1.5 million. The dataset covers a variety of object categories and complex scenes, which involve various challenging factors such as illumination changes, object occlusion, and rapid movement. It is widely used to evaluate the robustness and accuracy of tracking algorithms. We evaluate our method on the LaSOT test set and compare it with some mainstream trackers. As shown in Table 1, our method achieves a success rate of 72.6%, which is 1% and 1.2% higher than ARTrackV2 [37] and AQATrack [38], respectively.

**Table 1:** Comparison with other methods on LaSOT.

Method	Succ(%)↑	P_Norm_(%)↑	P(%)↑
Ours	**72.6**	**82.8**	**79.5**
ARTrackV2 [37]	71.6	80.2	77.2
AQATrack [38]	71.4	81.9	78.6
ARTrack [10]	70.4	79.5	76.6
SeqTrack [9]	69.9	79.9	76.3
GRM [25]	69.9	79.3	75.8
OSTrack [8]	69.1	78.7	75.2
AiATrack [5]	69.0	79.4	73.8
Mixformer [7]	69.2	78.7	74.7
TransT [4]	64.9	73.8	69.0
STARK [6]	67.1	77.0	72.2

↑ indicates higher scores are better.

GOT-10k is a large-scale benchmark dataset for general object tracking. It contains more than 10,000 video frame sequences, and the total number of frames is more than 1.5 million. The dataset covers a variety of object types and scenes, and contains a variety of complex challenges such as motion states and occlusions. Therefore, it is of great significance to test the generalization performance of tracking algorithms. According to the official requirements, we only use the training set of the dataset for model training, and evaluate our method through the official evaluation server. As shown in Table 2, our method achieves an AO score of 76.0%, which is 1.3% higher than that of SeqTrack [9].

**Table 2:** Comparison with other methods on GOT-10k.

Method	AO(%)↑	SR_0.5_(%)↑	SR_0.75_(%)↑
Ours	**76.0**	85.1	**74.7**
ARTrackV2 [37]	75.9	**85.4**	72.7
AQATrack [38]	73.8	83.2	72.1
SeqTrack [9]	74.7	84.7	71.8
GRM [25]	73.4	82.9	70.4
ARTrack [10]	73.5	82.2	70.9
OSTrack [8]	71.0	80.4	68.2
AiATrack [5]	69.6	80.0	63.2
Mixformer [7]	70.7	80.0	67.8
TransT [4]	67.1	76.8	60.9
STARK [6]	68.8	78.1	64.1

↑ indicates higher scores are better.

TrackingNet is a large-scale short-term object tracking benchmark. The dataset is divided into training and testing subsets, which contain more than 30,000 video sequences. We evaluate our method on its test set and use an online evaluation server to obtain performance metrics. As shown in Table 3, our method achieves a success rate of 85.0%, which is 1.2% and 0.8% higher than AQATrack [38] and ARTrack [10], respectively.

**Table 3:** Comparison with other methods on TrackingNet.

Method	Succ(%)↑	P_Norm_(%)↑	P(%)↑
Ours	**85.0**	**89.9**	**84.8**
ARTrackV2 [37]	84.9	89.3	84.5
HIPTrack [39]	84.5	89.1	83.8
AQATrack [38]	83.8	88.6	83.1
ARTrack [10]	84.2	88.7	83.5
SeqTrack [9]	83.3	88.3	82.2
GRM [25]	84.0	88.7	83.3
OSTrack [8]	83.1	87.8	82.0
AiATrack [5]	82.7	87.8	80.4
Mixformer [7]	83.1	88.1	81.6
TransT [4]	81.4	86.7	80.3

↑ indicates higher scores are better.

**Fig 2:**
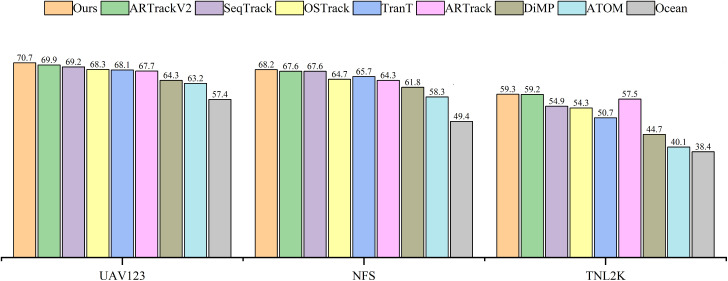
Comparison of AUC scores with other trackers on UAV123, NFS, and TNL2K datasets.

We also evaluate our method on three other tracking benchmarks, including UAV123, NFS, and TNL2K. UAV123 is a long-term tracking benchmark and comprises 123 video sequences captured from a drone perspective, which include diverse complex environments and challenges such as fast motion, viewpoint changes, and occlusions. NFS is a tracking benchmark consisting of 100 fast-motion video sequences. Similar to previous trackers, we use the 30fps version of NFS to evaluate our method. TNL2K is a large-scale dataset with natural language description, which contains 700 test sequences. Each sequence not only has visual data, but also has a natural language description. It is a challenging tracking benchmark. We compare our method with ARTrackV2 [37], SeqTrack [9], OSTrack [8], TransT [4], ARTrack [10], DiMP [40], ATOM [13], and Ocean [41]. As shown in Fig 2, our method achieves AUC scores of 70.7%, 68.2% and 59.3% on three datasets, respectively, which demonstrates excellent performance.

### 4.3. Ablation studies

In order to prove the effectiveness of the adaptive token division module, we use the GOT-10k dataset for comparative experiments. Both methods employ identical pre-training strategies, input resolutions, training data compositions, and evaluation protocols. As shown in Table 4, after introducing this module into the encoder, the model achieves an AO score of 76.0% with a slight loss in inference speed, which is 3.3% higher than that of the model without it.

**Table 4:** Comparison experiment of adaptive token division module.

Adaptive Token Division	GOT-10k			FPS
	AO(%)↑	SR_0.5_(%)↑	SR_0.75_(%)↑	
	72.7	82.0	70.3	55
√	76.0	85.1	74.7	50

↑ indicates higher scores are better.

Subsequently, to determine the optimal encoder layer for adaptive token division, we conduct three variant experiments: incorporating the module into all encoder layers (variant 2), into the last eight encoder layers (variant 3), and into all encoder layers except the first one (variant 4). The results of variant 2 in Table 5 show that it is not optimal to apply adaptive token division in the first encoder layer, because the initial token embeddings lack sufficient feature refinement. Specifically, the input tokens in the first encoder layer have only undergone linear projection. They have not yet been refined through attention-based feature aggregation or non-linear transformations. Their representations remain at a local, low-semantic patch level, which lacks global context and awareness of target structure. Making token division decisions based on such raw features is prone to interference, which often comes from local noise or background regions. As a result, it leads to unstable segmentation outcomes. This instability not only degrades the quality of cross-modal interaction, but also harms the target feature extraction in subsequent layers. Variant 4 has the best tracking performance. Therefore, in our method, we add the adaptive token division module to all encoder layers except the first layer.

**Table 5:** The ablation experiment on which layers of the encoder apply the adaptive token division module.

Variant	Layer	GOT-10k		
		AO(%)↑	SR_0.5_(%)↑	SR_0.75_(%)↑
1	None	72.7	82.0	70.3
2	1-12	73.7	82.8	71.4
3	5-12	74.9	84.1	73.3
4	2-12	76.0	85.1	74.7

↑ indicates higher scores are better.

Additionally, the separate processing of attention for different categories slows down the model. To address this issue, we introduce an attention masking strategy that unifies the computation. In our comparative experiments, the model with separate computation of attention runs at 50 fps, while our method with the masking strategy increases the speed to 65 fps.

## 5. Conclusion

In this paper, we propose a transformer-based visual tracking framework with adaptive token division. Our framework only uses encoders and decoders without any post-processing operations. In addition, this paper introduces an adaptive token division module in the encoder to achieve the better interaction between search tokens and template tokens. We employ an attention masking strategy to achieve parallel attention calculation between different token categories, and introduce Gumbel-softmax technique to promote end-to-end optimization of adaptive token division module. Experiments show that our method achieves excellent performance on the mainstream tracking benchmarks, which proves the effectiveness of our method in dealing with complex scenarios such as similar object interference.
